# Nondestructive 3D phenotyping method of passion fruit based on X-ray micro-computed tomography and deep learning

**DOI:** 10.3389/fpls.2022.1087904

**Published:** 2023-01-12

**Authors:** Yuwei Lu, Rui Wang, Tianyu Hu, Qiang He, Zhou Shuai Chen, Jinhu Wang, Lingbo Liu, Chuanying Fang, Jie Luo, Ling Fu, Lejun Yu, Qian Liu

**Affiliations:** ^1^ Britton Chance Center for Biomedical Photonics, Wuhan National Laboratory for Optoelectronics-Huazhong University of Science and Technology, Wuhan, Hubei, China; ^2^ MoE Key Laboratory for Biomedical Photonics, Huazhong University of Science and Technology, Wuhan, Hubei, China; ^3^ College of Tropical Crops, Hainan University, Haikou, China; ^4^ School of Biomedical Engineering, Hainan University, Haikou, China; ^5^ Sanya Institute of China Agricultural University, Sanya, China

**Keywords:** plant phenomics, micro-CT, passion fruit phenotypic traits, deep learning, nondestructive

## Abstract

Passion fruit is a tropical liana of the Passiflora family that is commonly planted throughout the world due to its abundance of nutrients and industrial value. Researchers are committed to exploring the relationship between phenotype and genotype to promote the improvement of passion fruit varieties. However, the traditional manual phenotyping methods have shortcomings in accuracy, objectivity, and measurement efficiency when obtaining large quantities of personal data on passion fruit, especially internal organization data. This study selected samples of passion fruit from three widely grown cultivars, which differed significantly in fruit shape, size, and other morphological traits. A Micro-CT system was developed to perform fully automated nondestructive imaging of the samples to obtain 3D models of passion fruit. A designed label generation method and segmentation method based on U-Net model were used to distinguish different tissues in the samples. Finally, fourteen traits, including fruit volume, surface area, length and width, sarcocarp volume, pericarp thickness, and traits of fruit type, were automatically calculated. The experimental results show that the segmentation accuracy of the deep learning model reaches more than 0.95. Compared with the manual measurements, the mean absolute percentage error of the fruit width and length measurements by the Micro-CT system was 1.94% and 2.89%, respectively, and the squares of the correlation coefficients were 0.96 and 0.93. It shows that the measurement accuracy of external traits of passion fruit is comparable to manual operations, and the measurement of internal traits is more reliable because of the nondestructive characteristics of our method. According to the statistical data of the whole samples, the Pearson analysis method was used, and the results indicated specific correlations among fourteen phenotypic traits of passion fruit. At the same time, the results of the principal component analysis illustrated that the comprehensive quality of passion fruit could be scored using this method, which will help to screen for high-quality passion fruit samples with large sizes and high sarcocarp content. The results of this study will firstly provide a nondestructive method for more accurate and efficient automatic acquisition of comprehensive phenotypic traits of passion fruit and have the potential to be extended to more fruit crops. The preliminary study of the correlation between the characteristics of passion fruit can also provide a particular reference value for molecular breeding and comprehensive quality evaluation.

## Introduction

Passion fruit (Passiflora edulis Sims) is native to Brazil and widely cultivated in warm areas of Asia, America, Australia and other regions (Corrêa et al., 2016; Teng et al., 2022). In recent years, there has been a continuous increase in its area and production in China (Chen et al., 2022). Passion fruit is popular for its captivating flavor, nutritional benefits, medicinal properties, and other economic value (Pongener et al., 2014). The center of the fruit contains many yellow, gelatinous pulp, and the juice also have a potent fragrance and is rich in sugar (Janzantti and Monteiro, 2017). Except as food, the whole fruit (pulp and pericarp) has sometimes been used in traditional medicines as a sedative or in therapies for the prevention of central nervous system disorders such as anxiety and insomnia (Sena et al., 2009; Miroddi et al., 2013). More than these, recent health-conscious trends have led to growing consumer demand for naturally derived colorants. The pericarp of passion fruit is an important raw material for natural colorants extraction (Kawasoe et al., 2021).

Nowadays, an important challenge in crop production and plant research is how to accelerate progress in breeding (Rahaman et al., 2015; Varshney et al., 2021). Breeding targets for crops can generally be divided into several broad categories: yield trait targets, quality trait targets, maturity period targets, tolerance targets to combat pests and pests, tolerance targets to environmental stresses, and fitness targets to protect the cultivated environment. Accurate acquisition and analysis of plant phenotypic traits are of great significance for improved breeding and functional gene mapping. In the past 20 years, the research of plant phenomics around the world has developed quite rapidly. Many non-destructive and high-throughput phenotyping methods have been widely used to automatically obtain plant phenotypic data, which are based on visible light imaging (Zingaretti et al., 2021), near-infrared imaging (Wang et al., 2014; Ambrose et al., 2016), infrared thermal imaging (Yang et al., 2020), hyperspectral imaging (Sun et al., 2022), X-ray computed tomography (X-ray CT), fluorescence imaging and magnetic resonance imaging (MRI) (van Dusschoten et al., 2016). Modern optical imaging technologies have achieved high-efficiency and non-destructive extraction of plant phenotypic traits (Sun et al., 2022). At the same time, the platforms carrying these optical devices have also made great progress, such as the greenhouse platform, vehicle platform, track platform, and UAV platform (Jin et al., 2021). These methods and platforms have realized the extraction and analysis of crop phenotypic traits and have been applied to the non-destructive testing of crops and to promote variety improvement and breeding.

However, most crop phenotypic research focuses on cereal crops such as wheat, rice, and soybean, and few research focuses on tropical crops especially passion fruit. An improved method has been proposed based on a Multiple Scale Faster Region-based Convolutional Neural Networks (MS-FRCNN) approach using the color and depth images acquired with an RGB-D camera to realize the detection of passion fruit in the actual orchard environment (Tu et al., 2020). There are also some scholars who use the visible light camera to obtain the color and other information of passion fruit, combined with physical and chemical descriptors digital image analysis, and then use the methods of principal component analysis and cluster analysis to predict the flesh quality of passion fruit. However, some indexes, such as pericarp thickness, still need to destroy the passion fruit sample for measurement, which will cause some measurement errors and be unfavorable to the inference of the final model (Jesus et al., 2022). Near-infrared (NIR) spectroscopy has also been applied to predict the total soluble solids, titratable acidity, and pulp content of passion fruit (Maniwara et al., 2019). The experimental result proved the feasibility of NIR spectroscopy for the evaluation of passion fruit quality.

The optical imaging technology in most of the above research can only obtain the shape and color information of the passion fruit surface without destroying the samples, but it cannot obtain its internal structural information. Benefiting from the rapid development of X-ray computed tomography (X-ray CT) with the function of perspective imaging of objects (Jenneson et al., 2003), it is gradually popularized from medical examination to other biological detection fields, including the imaging research of the internal structure of small animals (Bartling et al., 2008; Li et al., 2008; Ashton et al., 2015) and the imaging research of the internal structure of plants (Kotwaliwale et al., 2014). Researchers use this technical means to realize the non-destructive acquisition of the internal structure information of organisms. As for the application in fruits and vegetables, X-ray CT was used to evaluate the density and the water content in apples under varying moisture conditions (Tollner, 1992). Kim (Kim and Schatzki, 2001) used it to study the core breakdown development in pears and the segmentation and classification in hazelnuts (Khosa and Pasero, 2014). Recently, this technology has also been applied to the internal measurement of walnut (Bernard et al., 2020).

In order to distinguish plant samples and biological tissues, image segmentation technology has been widely used, which means classifying image pixels into different segments. Image segmentation technology can be divided into segmentation based on classical digital image processing technology (Cui and Zhang, 2018) and based on deep learning technology (Singh et al., 2018). In recent years, deep learning technology has been greatly developed. Due to its strong deep feature analysis ability and the convenience of end-to-end prediction, many researchers have applied the segmentation algorithm based on deep learning to the processing of plant images and achieved excellent results. Aich (Aich and Stavness, 2017) used deep learning architectures for initial segmentation and a convolutional network for leaf counting. Xiong (Xiong et al., 2017) designed a segmentation algorithm based on deep learning to achieve robust segmentation of rice panicle under different light environments and different growth states. Deep learning based segmentation technology were similarly applied in monitoring fruit growth (Fukuda et al., 2021), fruit detection and localization (Maheswari et al., 2021), detecting vascular bundles in computed tomography images of stem internodes (Du et al., 2022) and segmentation of major plant organs (Rawat et al., 2022). Overall, the thechnology based on classical digital image processing technology is easy to implement and interpretable but poorly generalizable. Overall, the segmentation technology based on deep learning is highly accurate and robust but requires large amounts of labeling data, and this process is labor-intensive.

Here, we present the development of a robust method that extracts the complete morphological traits of passion fruit for the first time using X-ray Micro-CT and deep learning. This method solves the problem that the traditional internal measurement method needs to destroy the sample, which could be laborious and error-prone, and realizes the nondestructive, accurate, and comprehensive measurement of passion fruit. More than that, we also analyze the correlation between the traits of passion fruit and propose a comprehensive evaluation method. Our results can be used as a reference for new breeding research. Biologists can cultivate better varieties by selecting the best germplasm as the basis of genetic improvement.

## Methods

### Plant materials and experiment design

In this study, 45 passion fruits were subjected to Micro-CT and manual measurements. These passion fruits were obtained in four separate batches, named PF041, PF042, Qinmi 9, and Tainong 1. Among them, PF041 and PF042 were cultivated in the Hainan University (Haikou City, Hainan province, China; 20.05°N, 110.3°E). Qinmi 9 and Tainong 1were cultivated in the passion fruit planting base in Sanya City (Sanya City, Hainan province, China; 18.33°N, 109.15°E), Hainan Province, China. It should be noted that PF042 and Qinmi 9 are generally considered to be the same variety. Due to the differences in their sources and growth stages, they were regarded as two types of samples in this paper.

After picking, all samples were transported to the laboratory in a sealed and refrigerated manner for the imaging experiment, which was divided into two parts: CT imaging experiments and manual measurement experiments. Subsequently, the fourteen phenotypic traits of passion fruit were calculated based on the CT imaging data. The artificially measured fruit width and length data were used as a reference to verify the reliability and robustness of the method in this paper.

**Figure 1:**
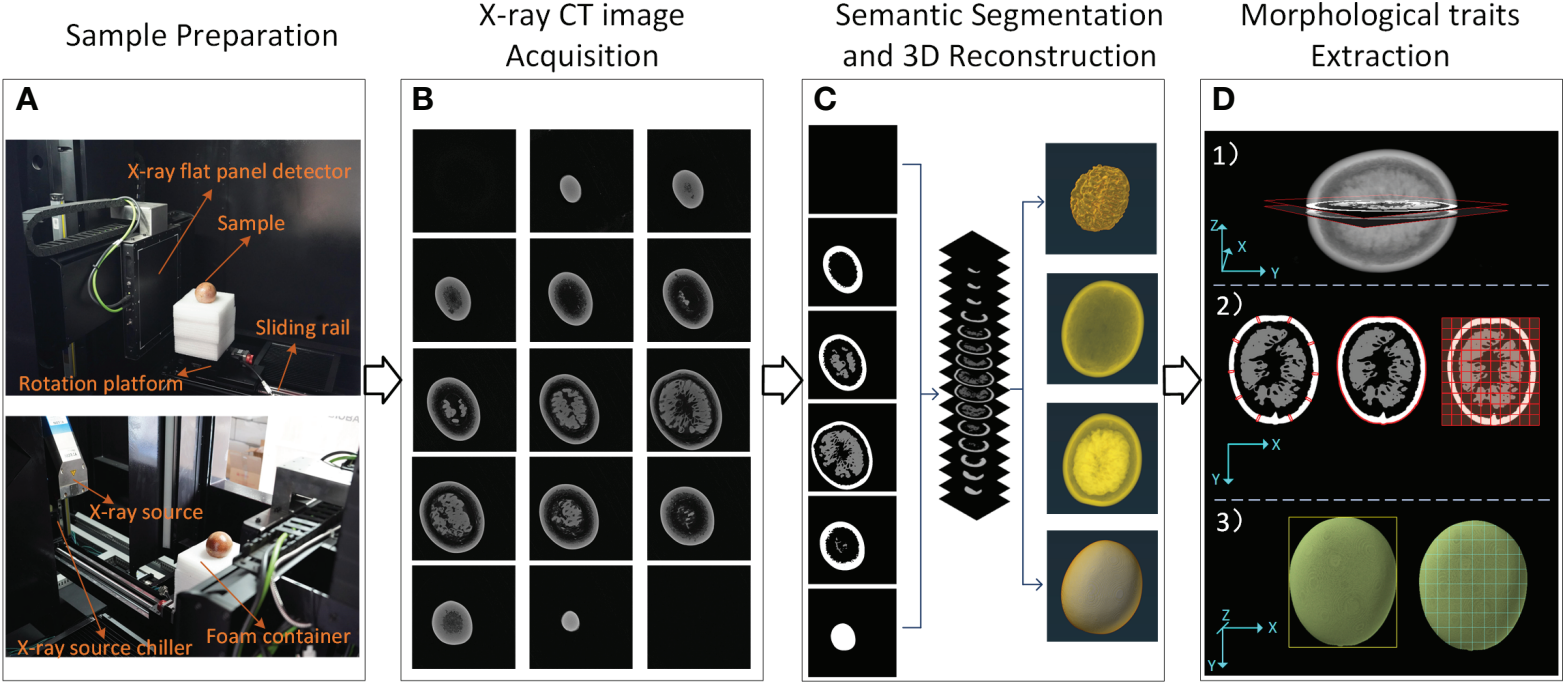
X-ray CT workflow of passion fruit measurements. **(A)** Composition of Micro-CT system and preparation of the sample. **(B)** Image acquisition. **(C)** Semantic segmentation and 3D reconstruction. **(D)** Morphological traits extraction.

The experimental workflow used in this paper is shown in **Figure 1**. As shown in **Figure 1**, the experimental process can be divided into four parts: (1) Sample preparation, (2) X-ray Micro-CT image acquisition, (3) Semantic segmentation and 3D reconstruction, and (4) Morphological traits extraction. In **Figure 1A**, the passion fruits were embedded in a flexible polyurethane foam sample holder (15 cm length × 15 cm width × 20 cm height) to keep the samples from any abrupt or slight movement during the scanning process in order to avoid producing distorted images. In **Figure 1B**, the X-ray Micro-CT scan data was reconstructed into several tomograms, and they were saved according to the scanning sequence (corresponding to the sample depth). In **Figure 1C**, the semantic segmentation method was performed on the basis of the tomograms. After that, the whole image was divided into pericarp, sarcocarp and background. In order to obtain more accurate extraction and quantification of their morphological traits, all the tomograms were sequentially stacked to reconstruct a three-dimensional image. In **Figure 1D**, an image processing pipeline was designed to measure the morphological traits of passion fruit based on three-dimensional images. Part of the traits was calculated based on the intermediate depth (**Figure 1D** 1)), such as pericarp average thickness, fruit longitudinal perimeter and fruit max cross-sectional area (**Figure 1D** 2)). Another part was calculated based on the whole data, such as fruit width, fruit width, fruit volume, and fruit surface area. The remaining part was calculated from the above traits. The details of each step will be introduced later.

### X−ray computed tomography imaging system specifications

**Figure 2:**
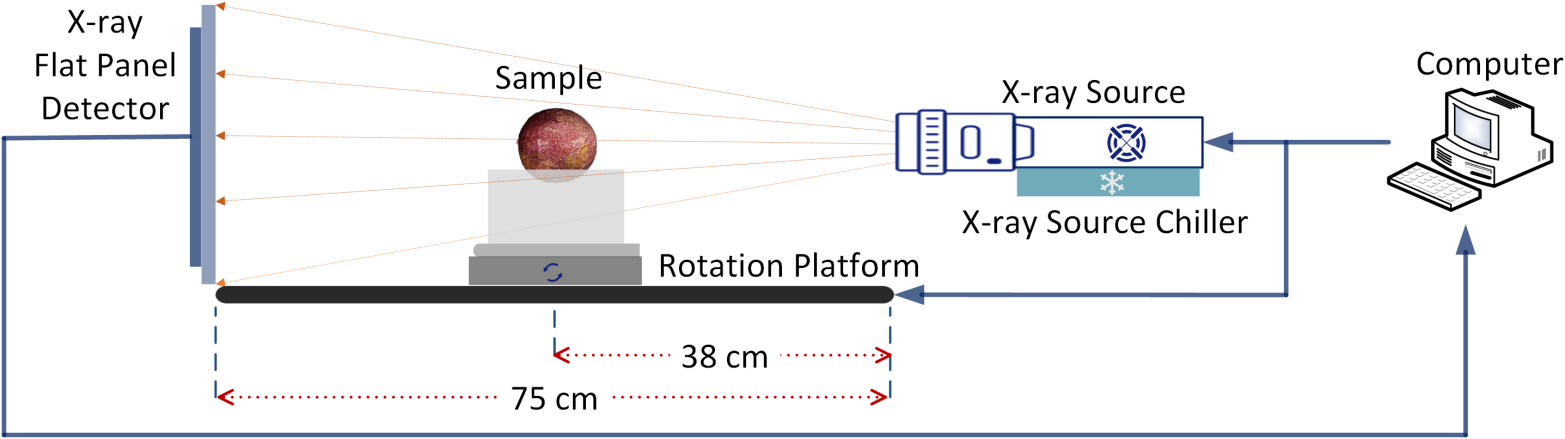
X-ray Micro-CT system schematic diagram.

The Micro-CT imaging system was developed to obtain CT projection images non-destructively. The system consists of six main elements: an X-ray source (Y.FXE-225.48, German), an X-ray source chiller (Nova600, OXFORD, UK), an X-ray flat panel detector (XRD3025N-G22-A, Varex, USA), a rotation platform (OMTOOLS 100B, Panasonic, Japan), a lead chamber, a computer (CPU i5-7700k, DELL), and a PLC controller (CP1H, OMRON Corporation, Japan). The schematic system diagram is shown in **Figure 2**.

As we all know, for a Micro-CT imaging system, choosing different tube currents and tube voltage will have a great impact on the imaging results (Stuppy et al., 2003). In order to achieve the best imaging effect of passion fruit, after a large number of confirmatory experiments, this paper set the tube current as 50 μA, the tube voltage as 100 kV, the distance from the ray source to the detector as 75cm, the distance from the ray source to the rotary table as 38cm, and the number of scanning frames as 360. Under this parameter, the time-consuming of each scanning effort by the Micro-CT system is about 10 min.

### Image acquisition and semantic segmentation

After the parameters of the Micro-CT system were set, each passion fruit sample was sequentially fixed in the designed flexible polyurethane foam container on the rotation platform (**Figure 1A**). In order to facilitate the traits calculation, uniformly place the passion fruit so that its longitudinal axis was parallel to the plane of the rotation platform as far as possible, and the top section was facing upwards. After the scanning was completed, the reconstruction algorithm was used to obtain the imaging results of each sample from the original data. It parsed them into several tomograms with a size of 2000*2000 pixels, which were saved in the TIF file format. This process takes about 5 minutes. Since the useless background occupied most of the pixels in each section, the region of interest (ROI) extraction algorithm was applied to each tomogram to reduce the background pixels while preserving the sample information. Such an operation could reduce the computation and make it more conducive for the deep learning network to obtain sample features. Finally, the size of each tomogram was reduced to 1000*1000 pixels.

**Figure 3:**
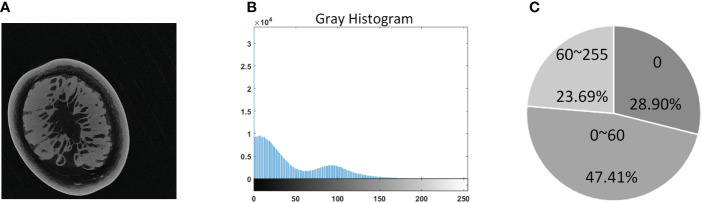
Grayscale distribution of tomogram images. **(A)** Example of a tomogram image. **(B)** The gray histogram. **(C)** Visualizes an approximate percentage of pixels in each cluster.

A single tomogram from the final imaging result was used as a representative to analyze image features in **Figure 3**. It can be seen from **Figure 3B** that the grayscale distribution of the tomogram was mainly divided into three intervals. The grayscale value of the pixels with a proportion of 28.9% was zero, and the grayscale between 0 and 60 accounted for 47.41% ratio; the number of pixels with a grayscale higher than 60 only occupied a ratio of 23.69%. Analyzing by combining the three illustrations in **Figure 3**, it could be found that the pixels with zero gray value were the background area, which will not interfere with the segmentation. The pixels with gray distribution between 0 and 60 were generally the inherent noise caused by the imaging system. At the same time, the sarcocarp and pericarp of passion fruit we were interested in were distributed in the range of 60-255. Significantly, there was no apparent gray difference between the sarcocarp and pericarp in **Figure 3A**.

Segmentation methods based on digital image processing technologies, combined with image filtering, morphological processing operators and other methods, and some prior knowledge, could show excellent segmentation performance on some tomography images (Hughes et al., 2017). Nevertheless, when applied to a large number of tomography image data with whole or even multiple passion fruit samples, it is very vulnerable to the changes in biological structure in the sample. Methods based on deep learning have been applied to biomedical image segmentation and achieved good results. However, using a deep learning model actually needs to prepare high-quality training sets in advance, which is very labor-intensive.

To solve the shortcomings when the two methods were applied to the segmentation of passion fruit tomogram images, this paper proposed a segmentation strategy that combined them: a label generation method based on digital image processing was designed to achieve segmentation of a series of images, and the part with higher accuracy in the segmentation results was picked as labels to be used to training deep learning model.

**Figure 4:**
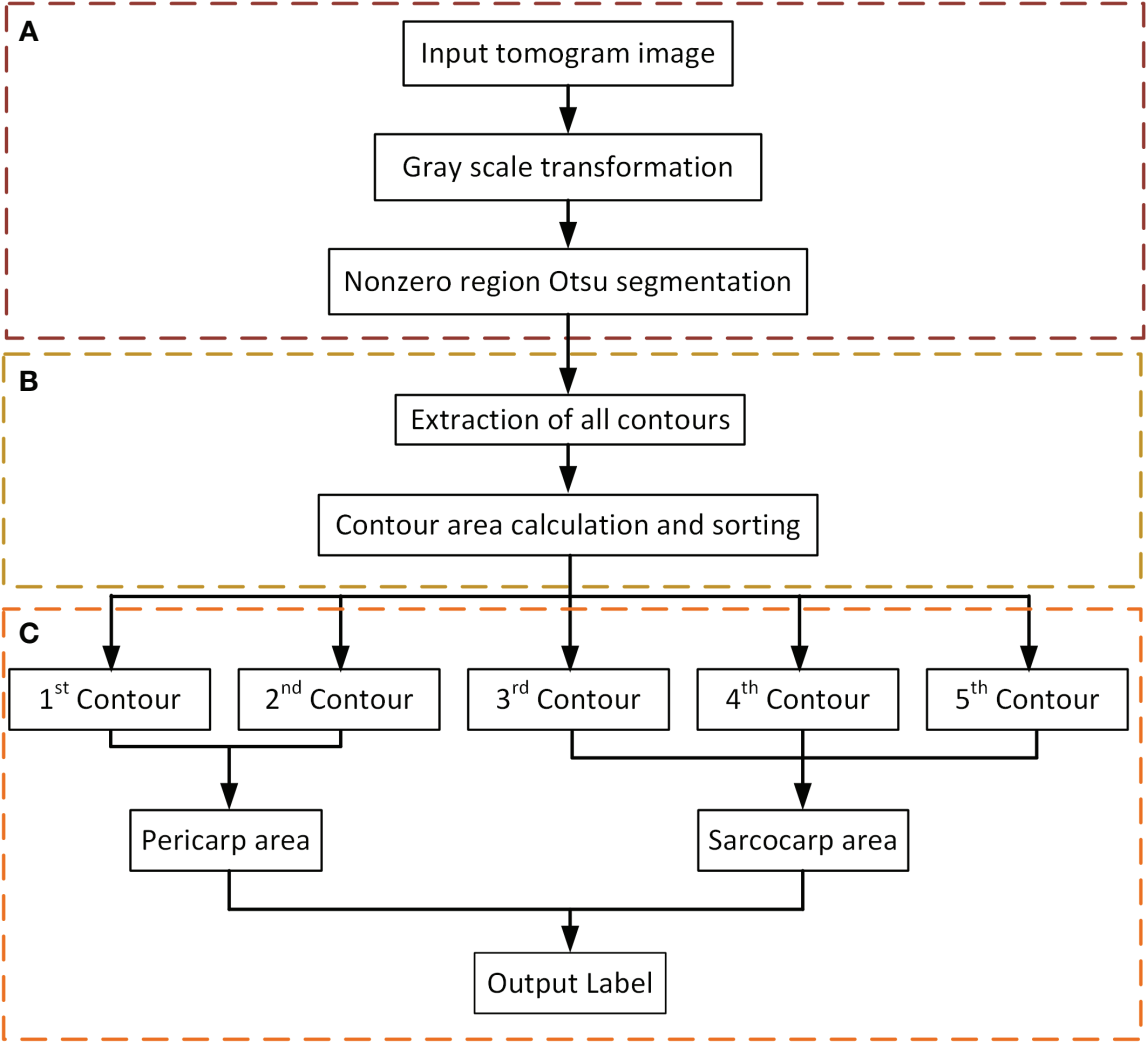
Flow of label generation method. **(A)** Image preprocessing. **(B)** Contour extraction. **(C)** Contour sorting and region segmentation.

The Flow of the label generation method designed in this paper is divided into three main steps (**Figure 4**): image preprocessing (**Figure 4A**), contour extraction (**Figure 4B**), contour sorting and region segmentation (**Figure 4C**). Firstly, take the tomogram image as input. The gray value of it was first transformed to suppress the background area with a low gray value to improve the image contrast. Then the Otsu segmentation algorithm was applied to the non-zero regions in the image (**Figure 4C**). The significance of this step was to calculate the threshold depending on the area of interest rather than the entire image, which would be more conducive to the segmentation of the sarcocarp and pericarp. Based on the previous step’s segmentation results, the image’s contours would be extracted (**Figure 4B**).

**Figure 5:**
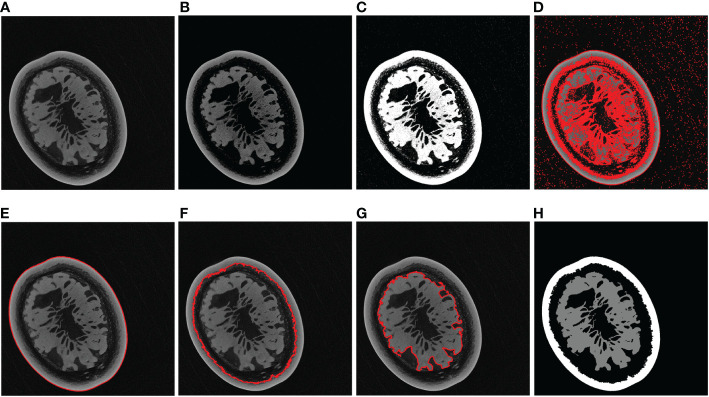
Label generation processing. **(A)** Example of a tomogram image. **(B)** After grayscale transformation. **(C)** Otsu segmentation result. **(D)** All contours. **(E)** Largest contour. **(F)** Second-largest contour. **(G)** Third-largest contour. **(H)** Label of the original image.

According to the actual situation (**Figure 5**), the contour with the largest enclosing area, the second-largest and the third-largest generally corresponded to the outer edge of the passion fruit sample (**Figure 5E**), the inner edge of the pericarp (**Figure 5F**), and the outer edge of the sarcocarp (**Figure 5G**). Of course, it cannot be ignored that the sarcocarp would be divided into several separate parts in the partial depth of the tomograph. At this time, the third-largest contour did not contain all the core parts, so we designed a judgment method: When the fourth-largest contour is greater than 25% of the third-largest contour, the sarcocarp part was the combination of the third and fourth contours. The sarcocarp of passion fruit was generally represented as a single-connected domain, double-connected domain and triple-connected domain in the tomogram image, so we only calculated the first five contours for now. Regarding the generated contour as a mask, the label (**Figure 5H**) was obtained by convolution of the mask and original image. In this way (**Figure 4C**), a large number of tomograms were processed, and even if there was a certain amount of data, the resulting labels were inaccurate due to differences in structure. Despite all this, it did not affect the generation of a sufficient number of accurate labels.

**Figure 6:**
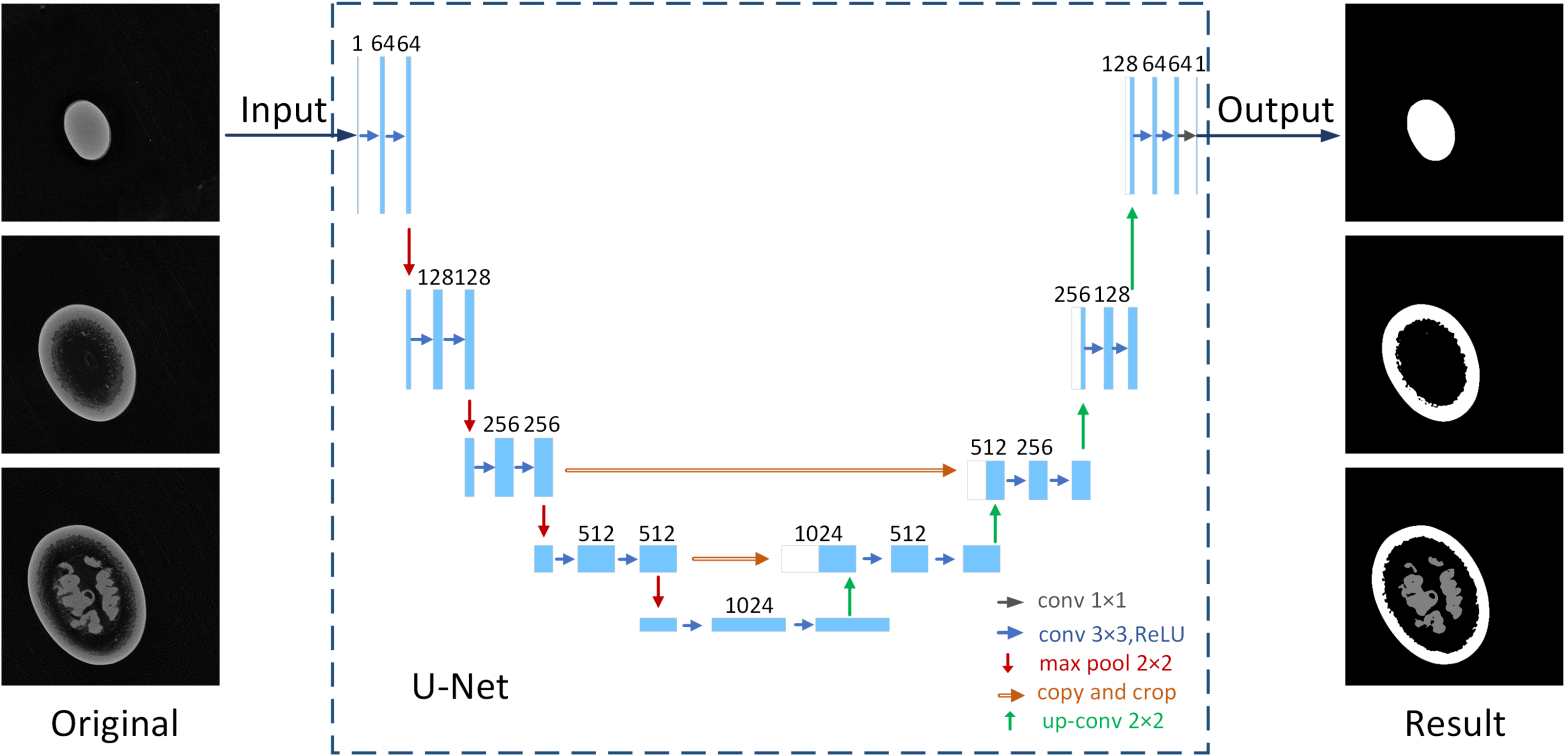
Application of U-Net convolutional model in tomogram image segmentation.

The U-Net convolutional model (**Figure 6**) was used for the semantic segmentation of passion fruit tomogram images. U-Net (Ronneberger et al., 2015) is a convolutional neural model which builds upon encoder-decoder architecture and is simply a hierarchical down-sampling convolutional layer followed by symmetric up-sampling convolutional layers, additionally feature maps from the encoder network are concatenated in the respective decoder part for the passage of semantic information. As an end-to-end network, it only needs to input images to get corresponding segmentation results after the network has been trained.

### 3D reconstruction and morphological measurement

According to the settings of Micro-CT system parameters and reconstruction algorithm parameters, the actual size of the single pixel of the tomogram is 0.1mm*0.1mm. At the same time, 1300 tomogram images correspond to the actual size of 13cm, which shows that the thickness of each tomogram map is also 0.1mm. By observing the actual imaging situation, it was found that 800 tomogram images were enough to include all the information of a single passion fruit sample. The other 500 tomogram images would be discarded to reduce the amount of calculation.

After these tomograms were stacked into three-dimensional images, the isotropic resolution data could be obtained without adjusting the step size. The size of the resulting 3D image was 1000*1000*800 pixels, and the size of a single voxel was 0.1mm*0.1mm*0.1mm. It would provide a benchmark for subsequent volume and area calculations.

Based on the segmented three-dimensional image of passion fruit, the fruit traits in **Table 1** could be calculated automatically in the designed image processing pipeline (**Figure 1D**). Volume is the complete connected-pixel count for each given sample (Hughes et al., 2017). The surface area was calculated by adapting a previously accurate method (Hu et al., 2020). The length and width of the passion fruit and the thickness of the pericarp were obtained by rectangle fitting and random sampling point distance measurement on several middle tomogram images (Zhu et al., 2022). All the traits of passion fruit were divided into three categories: passion fruit traits, sarcocarp traits and pericarp traits, which were the key indicators concerned in the scientific research, planting and production of passion fruit. The fruit traits include the length and width of passion fruit (also known as longitudinal diameter and transverse diameter), fruit surface area, fruit volume, max cross-sectional area (longitudinal) and fruit longitudinal perimeter.

**Table 1:** The classification and abbreviation of passion fruit traits.

Trait classification	Trait	Abbreviation	Unit
Passion Fruit Traits	Fruit width	FW	mm
	Fruit length	FL	mm
	Fruit surface area	FSA	mm^2^
	Fruit volume	FV	mm^3^
	Fruit max cross-sectional area	FMCA	mm^2^
	Fruit longitudinal perimeter Fruit length-width ratio Fruit shape index	FLP FLWR FSI	mm - -
Sarcocarp Traits	Sarcocarp volume	SV	mm^3^
	Sarcocarp content	SC	%
	Sarcocarp filling rate	SFR	%
Pericarp Traits	Pericarp volume	PV	mm^3^
	Pericarp content	PC	%
	Pericarp average thickness	PAT	mm

Moreover, the length-width ratio and fruit type index are calculated to evaluate the shape characteristics of passion fruit. The fruit length-width ratio (FLWR) is defined by (1) and the fruit shape index (FSI), which combines sphericity (Bernard et al., 2020) and the length-width ratio, is defined by (2). The closer the length-width ratio is to 1, the more coordinated the horizontal and vertical proportion of the passion fruit is. The closer the fruit shape index is to 1, the closer the whole fruit is to spherical.


(1) $$Fruit\;Length-Width\;Ratio=\;\frac{FL}{FW}$$

(2) $$Fruit\;Shape\;Index=\;\frac{\pi^{\frac{1}{3}}{\left(6FV\right)}^{\frac{2}{3}}}{FSA*FLWR}$$

At the same time, the pericarp volume, average thickness and sarcocarp volume of passion fruit could also be calculated, and then their proportion in the fruit volume would be calculated. Sarcocarp filling rate refers to the proportion of pulp in all components (including cavities and some low-density tissues) of passion fruit except the pericarp.

It should be noted that the workstation parameters during all the experiments are as follows: CPU: Intel i7-11700K @3.60 GHz, GPU: NVIDIA GeForce GTX3090, RAM: 64GB. The fully automatic calculation method for the phenotypic traits of passion fruit was developed based on the Python Language and OpenCV Library and run in PyCharm IDE. For an individual passion fruit, the process of image segmentation versus trait extraction takes approximately 100 seconds.

## Result

### Image data analysis and process of passion fruit

The pre-experimental results of Micro-CT reconstruction of a small number of passion fruit samples show that with the tube voltage set to 100 kV and the tube current set to 50 µA, the projection images with better contrast will be obtained. In order to reduce the influence of the low signal-to-noise ratio of cone-beam CT, 15 frames are collected and averaged within the same angle step.

**Figure 7:**
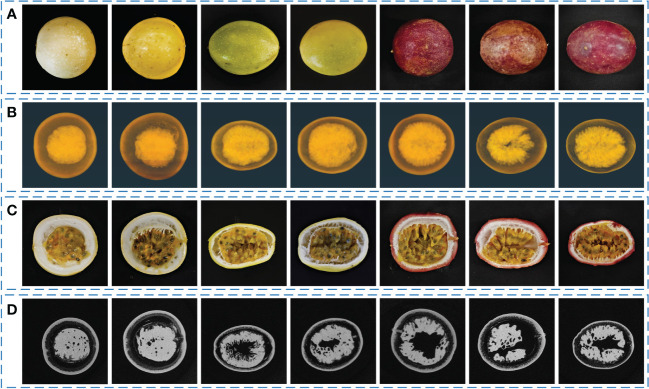
Seven morphologically representative passion fruit samples. **(A)** RGB images of samples. **(B)** 3D-reconstruction images of samples from Micro-CT. **(C)** RGB cross-sectional view of samples. **(D)** Tomogram images of samples from Micro-CT.

Seven morphologically representative passion fruit samples are selected from all the samples and shown in **Figure 7**. **Figure 7A** shows the RGB images of these seven samples. It can be seen that they are quite different in shape and color. Some samples have a shape that is close to spherical, while some samples are closer to ellipsoidal. **Figure 7B** shows the three-dimensional reconstruction results of passion fruit from the Micro-CT system. The three-dimensional images show that the sarcocarp and pericarp of passion fruit samples have a high X-ray absorption rate. The middle cavity and some low-density tissues are insignificant due to their low absorption rate. **Figure 7C** shows the cross-sectional view of each sample to observe the internal characteristics, from which it can be seen that there are also some differences in the distribution of various tissues within each sample. **Figure 7D** shows the tomogram images of samples from Micro-CT, which is also the basis for sarcocarp and pericarp segmentation and three-dimensional reconstruction.

**Figure 8:**
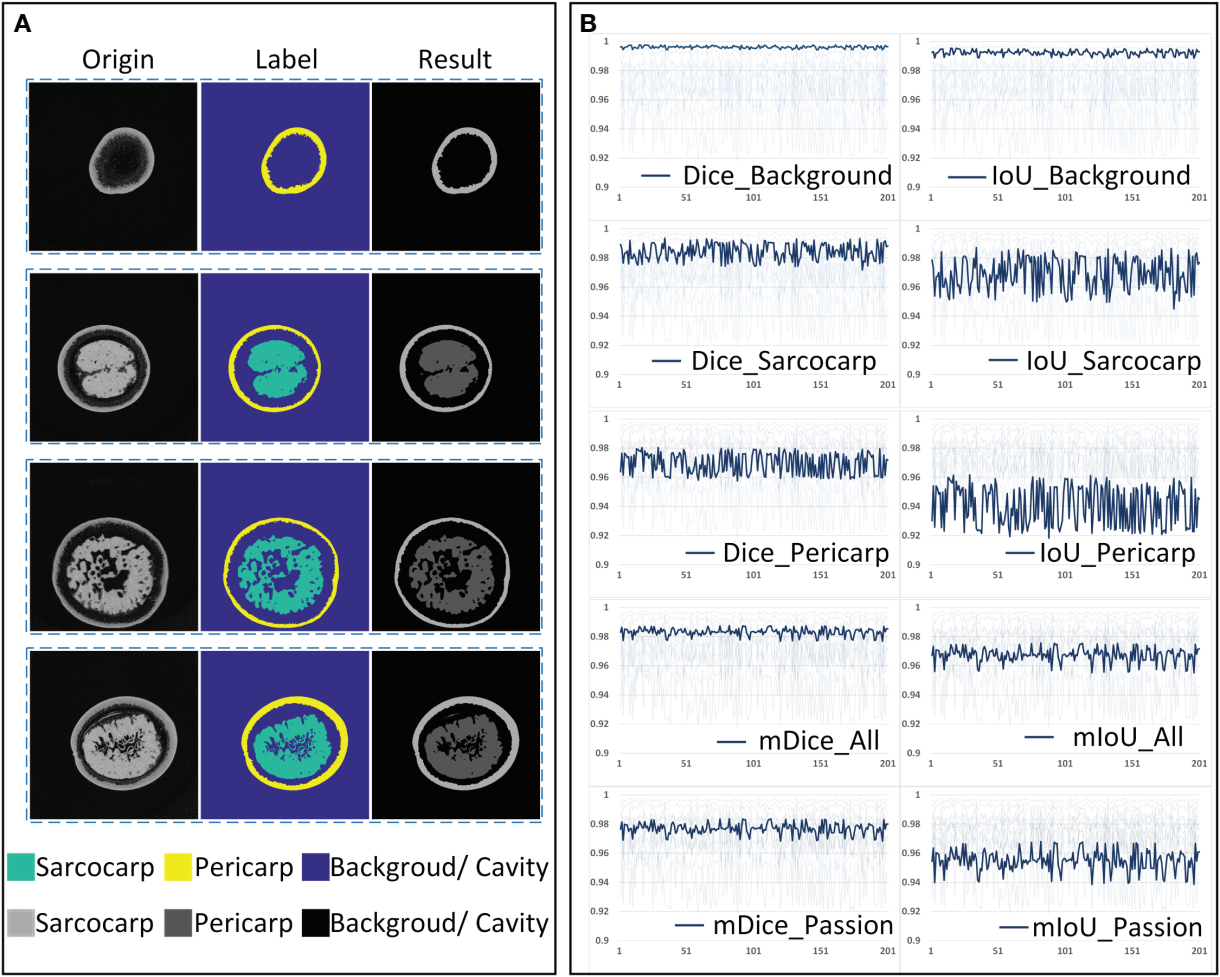
The accuracy analysis of the U-net segmentation model. **(A)** Original tomogram image, its label and segmentation result. **(B)** Dice and IoU of each category.

The segmentation of the tomogram was carried out under the U-Net segmentation model. As shown in **Figure 8A**, each tomogram was divided into three parts: sarcocarp, pericarp and background (including background and fibrous tissue with extremely low X-ray absorption rate). The data set used to train the segmentation network includes 2000 training sets and 400 verification sets. In order to verify the accuracy and robustness of the segmentation model based on U-Net, two hundred tomograms of different depths from different samples were randomly selected as the test sets, and then the manual labels and segmentation results were combined for verification. The segmentation accuracy was quantified using two parameters commonly used in segmentation algorithms, Intersection over Union (IoU) and Dice Coefficient (Dice). The batch size was set as four and RMSprop was used to optimize the model with an initial learning rate of 0.001. To match the receptive field of the network, the input images were scaled by 0.5. The U-net model was trained for a total of 200 epochs.

The quantitative and average indicators of each category are shown in **Figure 8B**. It can be seen that the mean Dice (mDice_All) and mean IoU (mIoU_All) of all three segmentation categories in the validation set reach about 0.983 and 0.971, respectively. After removing the background components, the mean Dice (mDice_Passion) and mean IoU (mIoU_Passion) of the passion fruit are about 0.975 and 0.955. Judging from the segmentation accuracy of each category, the Dice and IoU of the background are all above 0.990, the Dice and IoU of the sarcocarp are distributed around 0.985 and 0.972, and they are distributed around 0.973 and 0.945 for the pericarp. The result shows that the U-Net segmentation model has sufficient accuracy and reliable performance when it is used to segment passion fruit tomograms.

### Passion fruit morphological traits extraction

In order to obtain the results of three-dimensional reconstruction, the segmented results of all the tomogram images are stacked sequentially into a three-dimensional format, and then the three-dimensional digital model of each sample could be obtained. Fourteen traits, as shown in the **Table 1**, can be automatically calculated in the designed image processing pipeline. The descriptive statistics (mean, standard deviation minimum and maximum) of the fourteen traits in all samples are given in **Table 2**.

**Table 2:** Descriptive statistics of passion fruit morphological traits.

Morphological Trait	Mean ± SD	Range	Unit
Passion Fruit Traits			
Fruit width	61.05 ± 5.64	50.96 - 68.21	mm
Fruit length	65.88 ± 6.07	55.62 -73.19	mm
Fruit surface area	15733.21 ± 3345.44	10997.25 - 21726.56	mm²
Fruit volume	134524.15 ± 37998.12	87863.49 - 177060.82	mm³
Fruit max cross-sectional area	3119.32 ± 659.57	1827.85 - 3827.55	mm²
Fruit longitudinal perimeter	222.64 ± 25.35	184.32 - 308.23	mm
Fruit length-width ratio	1.08 ± 0.08	1.00 - 1.31	–
Fruit shape index	0.76 ± 0.12	0.49 - 0.87	–
Sarcocarp Traits			
Sarcocarp volume	36764.16 ± 9395.57	14466.47 - 52115.16	mm³
Sarcocarp content	28.01 ± 6.03	16.26 - 40.26	%
Sarcocarp filling rate	36.84 ± 7.47	19.09- 49.17	%
Pericarp Traits			
Pericarp volume	31553.56 ± 12518.67	13176.91 - 57428.58	mm³
Pericarp content	23.65 ± 7.78	12.96 - 56.41	%
Pericarp average thickness	2.69 ± 0.98	1.31 - 5.80	mm

It can be seen from **Table 2** that the average length and width of passion fruit samples in this experiment are 65.88mm and 61.05mm, the difference between samples is not more than 20mm, and the volume and surface area show significant differences. In addition, the average length-width ratio of the samples is 1.08, which indicates that most of the samples have relatively balanced dimensions in the horizontal and vertical directions. The fruit shape index also indicates this, but it can also be seen that there are still some flat and long individuals. The average proportion of the sarcocarp of passion fruit is 28%, and some samples reach 40%. Meanwhile, the average proportion of the pericarp is 23.65%, and some samples even exceed 50%. The average filling rate of the sarcocarp is 36.84%, which indicates that there are still a considerable number of cavities and other plant tissues in the fruit. The X-ray absorption rate by these tissues is extremely low, and the density is also very low.

**Figure 9:**
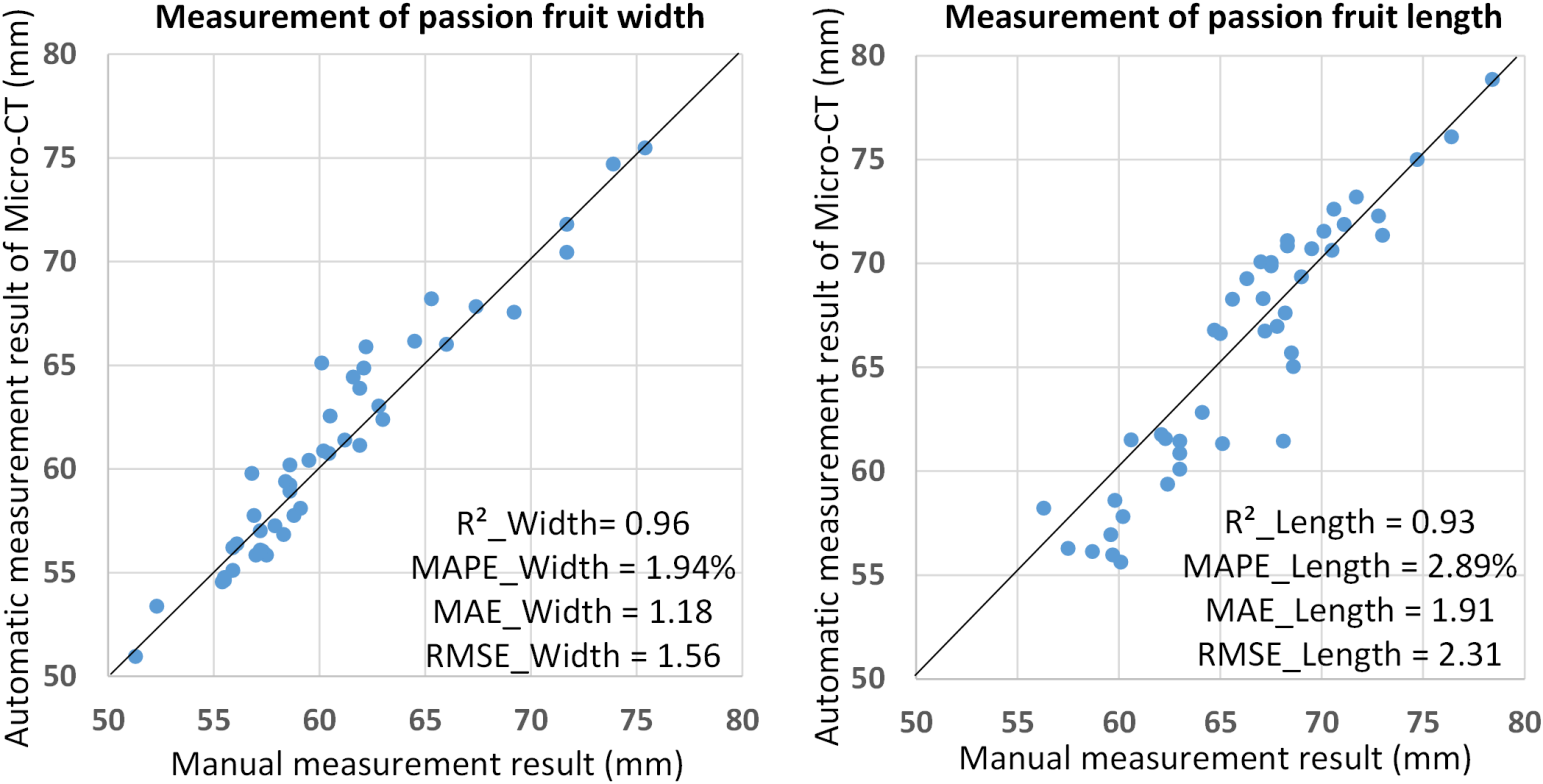
Automatic and manual measurement results of passion fruit length and width.

The manual measurement method was adopted to verify the accuracy of the automatic measurement method. As shown in **Figure 9**, the length and width of the passion fruit were manually measured. The results show that the R^2^ coefficients of the manual measurement and the automatic measurement method in the measurement of width and length are 0.96 and 0.93, the Mean Absolute Percentage Error (MAPE), Mean Absolute Error (MAE), and Root Mean Squared Error (RSME) of width are 1.94%, 1.18 and 1.56. As for length, they are 2.89%, 1.91 and 2.31. That is, for a passion fruit sample with a length and width of about 6cm, the error of the automatic measurement result is within 2mm. Such results can fully prove the reliability and accuracy of the automatic measurement method.

## Correlations and validation

### Pearson correlation matrix for passion fruit morphological traits

**Figure 10:**
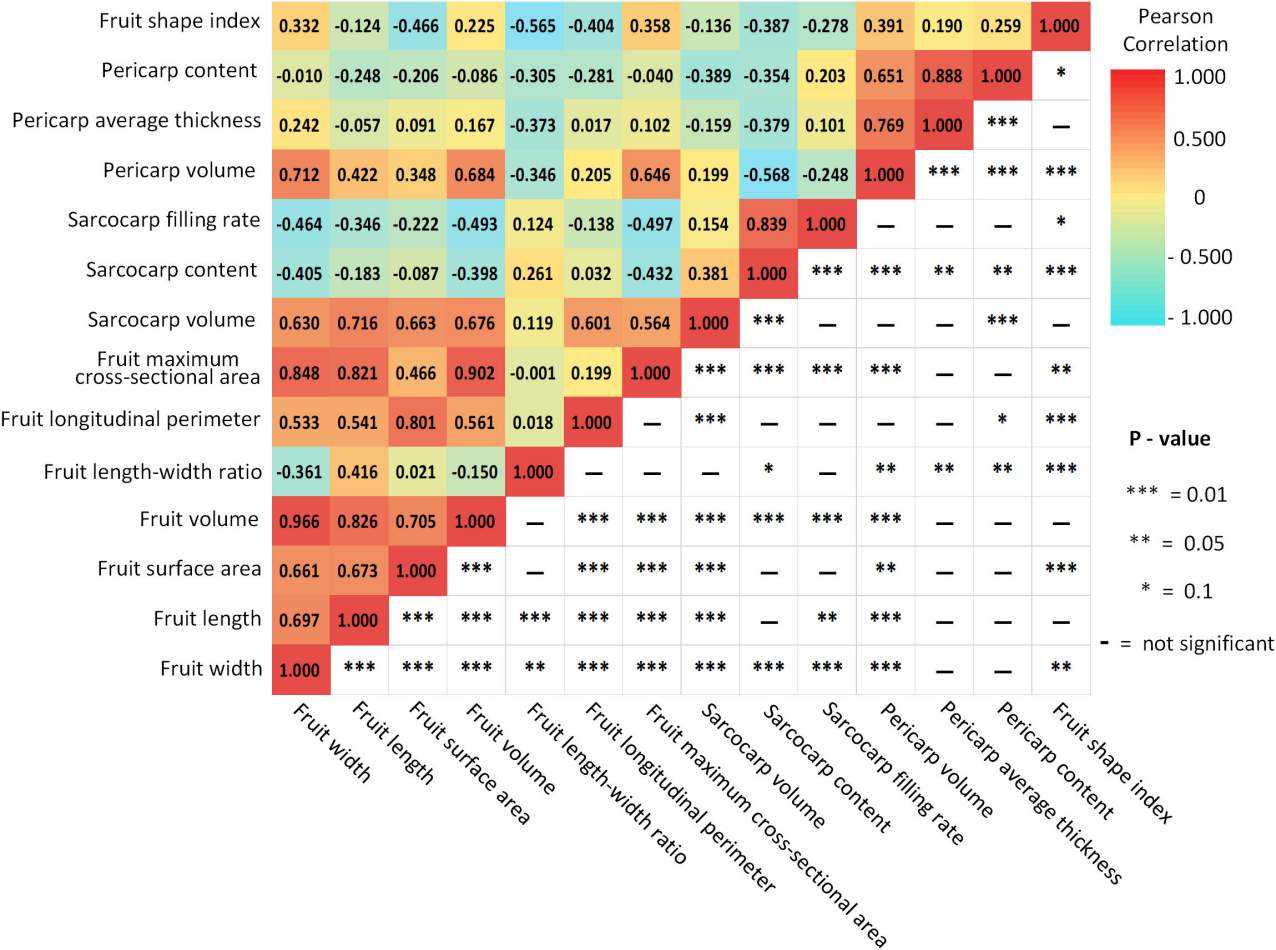
Pearson correlation matrix for passion fruit morphological traits.

Using the Pearson correlation coefficient, it is found that there is a specific correlation between these traits. According to **Figure 10**, it is evident that there is a strong positive correlation between the volume, surface area, length and width of passion fruit (0.661-0.966, P value 0.01), which follows the physical law. The max cross-sectional area strongly correlates with length, width and volume (0.821-0.902, P value 0.01). The longest girth shows the strongest correlation with fruit volume (0.801, P value 0.01), which is reasonable because neither length nor width alone can determine the final size of this feature. The volume of the sarcocarp and pericarp are positively related to the volume of the whole fruit. However, the general correlation (0.684, 0.676) is caused by differences between individuals and varieties. The filling ratio of fruit sarcocarp is highly correlated with its volume (0.839, P value 0.01). It shows a certain degree of negative correlation with length, volume and max cross-sectional area (-0.464, -0.493, -0.497), which indicates that the larger the size of the fruit, the lower the plumpness of fruit sarcocarp. At the same time, it can be noted that the proportion of the pericarp and its thickness show a strong correlation (0.888, P value 0.01), and the thickness of the pericarp is also strongly correlated with the volume of the pericarp (0.769, P value 0.01). At the same time, there is no significant correlation between the volume of the pericarp and the sarcocarp. The fruit shape index shows a negative correlation with the length-width ratio and surface area (- 0.565, - 0.466). When the length-width ratio is smaller, the fruit will be closer to the sphere, and the fruit type coefficient will be smaller. For three-dimensional objects, the surface area of the sphere is the smallest under the same volume. In this way, the conclusion of the analysis is consistent with the physical reality. In molecular breeding, comprehensive information is needed to support the analysis of the relationship between traits and genes. The specificity correlation among various characters of Passion Fruit can provide new reference information for breeders.

### Principal component analysis for passion fruit morphological traits

In the passion fruit industry, manufacturers and consumers have special expectations. For example, manufacturers hope that the proportion of sarcocarp and pericarp in fruits will be higher to obtain higher economic benefits. At the same time, consumers are more inclined to buy fruits with a more round and beautiful appearance and larger volume. Using these traits data for principal component analysis can provide some references for a comprehensive evaluation of passion fruit sample quality.

**Figure 11:**
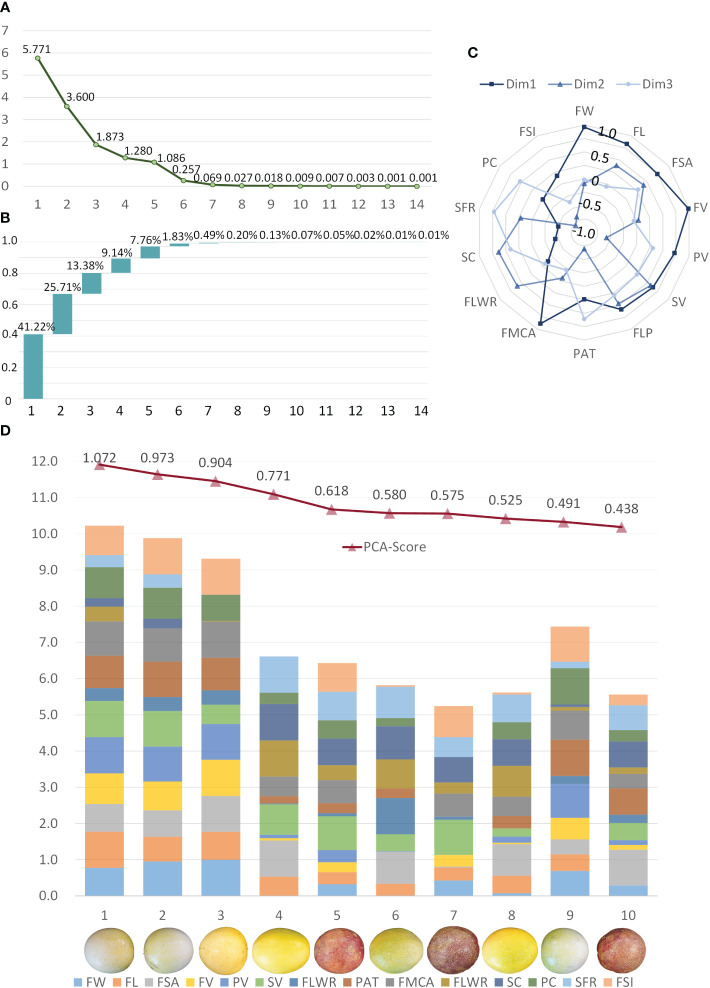
Principal Component Analysis using the passion fruit samples and the 14 traits quantified. **(A)** The characteristic root of the 14 components in PCA. **(B)** Scree plot of the percentage of components explained by all dimensions. **(C)** The contribution of each component to the first three dimensions. **(D)** Top ten samples of comprehensive quality evaluation and normalized expression of their 14 components.

**Figure 11A** is a gravel map drawn according to the data variation of the principal components. The eigenvalues tend to flatten after the third principal component. Combined with the variance interpretation rate of each principal component in **Figure 11B**, the first three dimensions of PCA explain 80.31% of the total variance. **Figure 11C** shows the contribution of each component in the first three principal dimensions. Dimension 1 corresponds to the morphometric traits (FW, FL, FMCA), the volumes (FV, PV, SV), and the fruit surface area. Dimension 2 is linked to the related traits of sarcocarp, such as volume and proportion. Dimension 3 focuses on the content of the sarcocarp and pericarp, which can be abstracted to describe the proportion of valuable parts in the fruit. According to the weight calculation results of each component of the principal component analysis, the sample individuals can be comprehensively evaluated by these fourteen traits. The top ten samples have scores ranging from 0.438 to 1.072 as shown in **Figure 11D**. Among all samples, these samples are in the head position in terms of size and volume. In order to better represent the contribution of each trait in the sample, all the trait data were normalized within the range of these ten samples, and finally, the stacked bar graph shown in **Figure 11D** was formed. The samples with the top three scores are among the best because of their large size, regular shape and prominent fruit volume, which coincide with the shopping philosophy of consumers. The remaining samples also have their own outstanding advantages in sarcocarp volume, sarcocarp filling rate and other traits. The comprehensive quality evaluation method based on principal component analysis can help producers and breeders understand the quality of samples more comprehensively and objectively. It can provide support for molecular breeding research aimed at obtaining better varieties.

## Discussion

### Advantages and disadvantages of Micro-CT system for passion fruit phenotype traits measurement

Nowadays, the Micro-CT system has been widely used in the medical field. Because of its nondestructive detection ability, it is also widely applied in crop research. This study applies this technology to the traits measurement of passion fruit for the first time. Fourteen phenotypic traits, such as fruit volume, length, width, sarcocarp volume and pericarp thickness, are obtained without damage and with high accuracy. It has prominent advantages over other traditional methods. First of all, it is a nondestructive measurement method. When using traditional methods to measure the internal traits of fruit, such as sarcocarp volume and pericarp thickness, it is necessary to cut the sample, which will lead to the rupture, deformation, and loss of some tissues. In particular, the flesh of passion fruit is very easy to liquefy, introducing specific errors in measuring passion fruit.

Moreover, the damaged fruits will no longer have any use value. After nondestructive measurement methods process the samples, the physiological and biochemical status of the samples will not be affected, which can continue to be used for in-depth research in gene metabolism and other fields. Secondly, the Micro-CT system can provide a three-dimensional digital image model of the sample, which is difficult for traditional sensors to provide. For example, although the structured light camera can provide a three-dimensional fruit model, it can not obtain internal traits. According to the three-dimensional digital model, the sample traits can be fully automatically extracted, effectively avoiding the subjective bias in manual measurement methods. The defect of the Micro-CT based measurement method mainly lies in that the physical density and the absorption rate of the X-ray of each biological tissue are different, and the response intensity reflected on the image is determined by both of them. In addition, it is affected by noise, which often leads to the situation that the gray levels of different tissues are close or some low-density tissues are difficult to distinguish from the air. The main parameters of the Micro-CT system, such as tube voltage and current, need to be adjusted before the experiment to obtain the best imaging effect.

The method studied in this paper has been well applied to passion fruit, but its significance is far more than this. These techniques and calculation methods can also be applied to other tropical fruits, such as coconut and pitaya. The reason is that there are often some density differences between the tissues of these tropical fruits, and the mass attenuation coefficients are also different, which will be reflected in the final imaging results. When calculating various characters based on the imaging results, because they all have inner and outer wrapping structures similar to Passion Fruit, the method used in this paper can be applied to other tropical fruits with only a few modifications. In the long run, because of their nondestructive characteristics, Micro-CT-based measurement methods have the potential of dynamic monitoring and character mining, which can continuously monitor the same sample in different growth or decay cycles and establish a complete growth or decay model. At the same time, this measurement method can mine more meaningful new traits, which is essential in promoting the research of plant development mechanisms and pathological influences. It is beneficial to cultivate new varieties, improve yield and quality, and has important practical significance in promoting the development of the agricultural economy.

### Deference of segmentation based on traditional digital image process and deep learning

In image processing, especially in image segmentation, the primary methods can be divided into algorithms based on traditional digital image processing (DSP) and deep learning technology. In comparison, the traditional digital image processing methods are more straightforward and faster, while the methods based on deep learning have more vital generalization ability and better performance.

For the tomogram image segmentation of passion fruit, we used the standard structural features of the samples as prior knowledge to design a feature extractor. Finally, we achieved fast image segmentation based on DSP. When applied to a large amount of data, only about 30% of the data showed excellent results. In the remaining images, the segmentation effect was poor due to structural and grayscale differences, and it was not easy to directly use it for subsequent statistics and calculations. On the other hand, the deep learning method needs to manually mark the image before using it. It takes about 2 minutes to mark a 2000*2000 pixels Passion Fruit tomogram, and it is easy to introduce personal subjective bias in the marking result.

Because of these situations, this paper combined the two kinds of methods. In order to automatically and quickly generate labels, a segmentation algorithm based on DSP was designed to process a certain number of original images, and the one with the better effect was selected as the training set. It was used for network training of subsequent deep learning, and finally, the trained network was used to predict all the data to obtain the final segmentation result. The experimental results showed that the designed method for generating training set labels was reduced from the artificial 120 seconds/frame to 2 seconds/frame, and the accuracy was very little different from the manual labeling. The method in this paper performed more objectively on some ambiguous pixels decisive. The accuracy of the final network prediction results also reached a high level. Our method can also be extended to other crops, and the process is relatively simple.

## Conclusions

This study presented a phenotype traits measurement method of passion fruit based on Micro CT and deep learning technology, which realized the nondestructive automatic and rapid extraction of fourteen phenotypic traits of passion fruit. Based on the phenotypic data of several samples, Pearson correlation analysis was carried out to mine the possible internal correlation, and the comprehensive quality of passion fruit was evaluated by combining principal component analysis. The experimental results showed that our proposed method not only filled the gap in the phenotypic measurement of passion fruit but also was a potential method for other species with similar structures. It could play a vital role in the future breeding improvement and industrial production.

## Data availability statement

The raw data supporting the conclusions of this article will be made available by the authors, without undue reservation.

## Author contributions

YL and LY designed the research, performed the experiments, analyzed the data and wrote the manuscript. RW, TH, QH, ZC, JW, LL, CF, JL and LF helped to perform the experiments. LY and QL supervised the project and helped to design the research. All authors contributed to the article and approved the submitted version.
